# Digital twins for land-based aquaculture: A case study for rainbow trout ( *Oncorhynchus mykiss*)

**DOI:** 10.12688/openreseurope.14145.2

**Published:** 2023-11-28

**Authors:** Adriano C. Lima, Edouard Royer, Matteo Bolzonella, Roberto Pastres

**Affiliations:** 1 Department of Environmental Sciences, Informatics and Statistics, Università Ca’ Foscari Venezia, Campus Scientifico, via Torino 155, Mestre, Venice, 30172, Italy; 2 Atlantic International Research Centre (AIR Centre), Parque de Ciência e Tecnologia da Ilha Terceira, Angra do Heroísmo, Azores, 9700-702, Portugal; 3 Bluefarm s.r.l., Centro Vega ed. Pegaso, via delle Industrie 15, Marghera, Venice, 30175, Italy

**Keywords:** Aquaculture, Precision Fish Farming, digital twin, trout, recirculating aquaculture system, land-based aquaculture, bioenergetic model

## Abstract

The virtual, digital counterpart of a physical object, referred as digital twin, derives from the Internet of Things (IoT), and involves real-time acquisition and processing of large data sets. A fully implemented system ultimately enables real-time and remote management, as well as the reproduction of real and forecasted scenarios. Under the emerging framework of Precision Fish Farming, which brings control-engineering principles to fish production, we set up digital twin prototypes for land-based finfish farms. The digital twin is aimed at supporting producers in optimizing feeding practices, oxygen supply and fish population management with respect to 1) fish growth performances; 2) fish welfare, and 3) environmental loads. It relies on integrated mathematical models which are fed with data from in-situ sensors and from external sources, and simulate several dynamic processes, allowing the estimation of key parameters describing the ambient environment and the fishes. A conceptual application targeted at rearing cycles of rainbow trout ( *Oncorhynchus mykiss*) in an operational in-land aquafarm in Italy is presented. The digital twin takes into account the disparate levels of automation and control that are found within this farm, and considerations are made on preferential directions for future developments. In spite of its potential, and not only in the aquaculture sector, the development of digital twins is still at its early stage. Furthermore, Precision Fish Farming applications in land-based systems as well as targeted at rainbow trout are novel developments.

## Plain language summary

A digital twin is set up for a rainbow trout ( *Oncorhynchus mykiss*) aquaculture farm located in northern Italy. The farm is equipped with large-scale basins, and are supplied by freshwater from the neighbouring Sarca river. Generally speaking, digital twins are virtual, digital representations which mirror and are connected to real objects, enabling real-time and remote management, as well as the reproduction of real or forecasted scenarios. This is a pioneer application aimed at supporting producers in optimizing feeding practices, oxygen supply and fish population management with respect to 1) fish growth performances; 2) fish welfare, and 3) environmental loads. The digital twin relies on sensors, Big Data, IoT (Internet of Things) and predictive mathematical models. In spite of its potential, and not only in the aquaculture sector, the development of digital twins is still at its early stage. Furthermore, Precision Fish Farming applications in land-based systems as well as targeted at rainbow trout are novel developments.

## Introduction

Industry 4.0 and sustainable farming are key factors for advancing aquaculture production and to respond to the growth vision of the EU aquaculture industry of reaching the provision of 4.5 million tons of sustainable food annually by 2030 ^1^. Currently, the reduction in cost of monitoring devices and the increase in computation power has been revolutionizing the management of aquaculture farms. Originally limited mostly to cage cultures of salmon (e.g., *Salmo salar*) farms ^2^, Precision Fish Farming (PFF) practices have been recently implemented in inland cultures of Rainbow trout ( *Oncorhynchus mykiss*), Carp ( *Cyprinius carp*), European seabass ( *Dicentrarchus labrax*) and Gilthead seabream ( *Sparus aurata*) ^3, 4^. The framework of PFF has also been extended to Precision Shellfish Farming (PSF), with implementations targeted at oyster ( *Magellana gigas*) and mussels ( *Mytilus edulis*, *Mytilus galloprovincialis*) ^3^. The implementation of the PFF approach aims at improving accuracy, precision, and repeatability in farming operations by delivering reliable decision-making support tools to farmers ^2^. PFF brings control-engineering principles to fish production, to improve the management of fish farms in terms of feed conversion, fish welfare and reduction of wastes. To do so, PFF relies on 1) large quantitative datasets provided from sensors which feed 2) data driven and mechanistic models and algorithms which finally supply 3) decision-supporting tools and smart management systems based on the Internet of Things (IoT) ^5^. Although the scope of PFF is well defined, its implementation is still in its early stages, as it requires investments in farm instrumentation and development of specific modelling approaches and software. This is particularly challenging for small and medium-sized enterprises (SMEs), where investment capacity is usually limited and the farm management phases to be integrated within PFF, i.e., 1) observing, 2) interpreting, 3) deciding and 4) acting ^2^, still rely essentially on experience-based interpretation and decision-making, and manual actions.

In this work, a digital twin applied to finfish inland aquaculture farms is proposed, based on the PFF management phases, and envisaging the use of sensors, Big Data, Internet of Things (IoT) and predictive mathematical models. Essentially, digital twins are virtual, digital representations which mirror and are connected to real objects ^6, 7^, enabling real-time and remote management, as well as the reproduction of real or forecasted scenarios. For comprehensive reviews on the concept of digital twin, the reader can refer to Grieves ^8^, Liu *et al*. ^9^, Verdouw *et al*. ^10^. For aquaculture, related concepts addressed extensively by this sector include 1) Virtual technology, defined as the means by which conceptual models can be made more formal and tested against reality ^11^, and 2) farm-scale production models, which integrate and simulate diverse farm operations in order to assess production and environmental loads ^12^. Beyond incorporating such concepts, digital twins in aquaculture, as in applications in other fields, generally foresees a real-time and remote connection between the real and virtual counterparts, with IoT being a key technology ^13^, along with non-internet technologies. For instance, advances in production efficiency, quality control and sustainable practices in agriculture and aquaculture systems, require non-internet technologies such as mechanisation of farm operations, advances in feed technologies, implementation of environmental-friendly practices and pathogen management ^14, 15^.

Despite its potential and notable advances, the development of Digital Twins is still at its early stage, as simulations applied along the operational phase of an implemented system have received little attention compared to those based on theoretical and static models aimed at verifying, validating and optimizing a system in its planning stage ^9^. Furthermore, most of the research works on digital twins were either on a theoretical level only, or implemented in laboratory ^9^. This paper is tailored at filling these gaps in the aquaculture sector, by combining typical approaches for digital twin frameworks with state-of-the-art PFF techniques targeted at a commercial pilot site. A conceptual digital twin which mirrors the cultivation of fishes in inland cultivation basins is demonstrated, employing an approach based on the quantitative processing of environmental data and bio-responses that makes it possible to optimise the feeding, oxygen supply, water quality and fish biomass transfers. A case study based on a real operational Rainbow trout ( *Oncorhynchus mykiss*) farm is presented.

## Methods

The physical objects and the production activities to be mirrored by the digital twin must be defined precisely and must suitably fit into digital twin frameworks, which in this study are built up based upon fundamentals from industry and agriculture. Thereby the methodology incorporates the following actions: 1) framing of aquacultural activities to be incorporated into the digital twin framework, 2) development of a typology for the digital twin according to the desired level of prediction and control, 3) definition of the specific physical components that will be emulated by the digital twin, and 4) definition on how the physical object is transformed into the digital counterpart in terms of variables and modelling.

This section also introduces aspects which are beyond the scope of the target conceptual application, in order to provide a broad contextualization of the adopted solutions and to introduce possibilities for further developments.

### Target activities and product lifecycle

The digital twin envisaged in this study incorporates the four management phases of PFF, i.e., observing, interpreting, deciding and acting ^2^, with a target on the controls of feed, oxygen and biomass along the fish growth from fingerlings to the commercial weight. Fish fertilization and fish early life development stages will be out of scope, along with other boundary activities such as decisions on quantities to be seeded, external logistics, market dynamics, costumer-supplier fluxes, business plans, and economic analysis.

There are digital twins addressing all lifecycle phases of industrial products, namely 1) design, 2) manufacturing (production), 3) service and 4) retire ^16, 17^. Among these, our target twin falls specifically into the production phase, which concerns the internal logistics of production systems.

In the context of finfish aquaculture, the design phase would typically correspond to the planning of a plant or of a forthcoming fish rearing cycle, whereas the service phase would comprise external logistics operations, such as those before fish seeding and after harvesting of fish (e.g., distribution of goods from suppliers and to costumers), and the retire phase would describe operations associated with decommission of the plant or of some its components.

### Digital twin levels

Based on previous works, Verdouw *et al*. ^10^ categorized digital twins into six typologies, referred as 1) imaginary, 2) monitoring, 3) predictive, 4) prescriptive, 5) autonomous and 6) recollection. Disparate typologies may coexist in the same twin, such that the typologies may be referred as the different levels of a digital twin.

Imaginary digital twins representing objects that have not yet been physically constructed, and recollection digital twins maintaining information from physical objects which are no longer operational or no longer exist, may correspond respectively to the above-mentioned design and retire phases of the product lifecycle. The remaining four typologies, or levels, correspond to the production and service phases of lifecycle, and are the typical focuses of digital twins. These levels, i.e., monitoring, predictive, prescriptive and autonomous can be, to a certain extent, roughly fitted into the observing, interpreting, deciding and acting management phases of PFF, respectively.

For an operational finfish farm, a monitoring digital twin would typically characterize the real, current state, and the dynamics of a fish cultivation basin, based on real-time data concerning water quality variables, such as water temperature, pH and concentrations of dissolved oxygen (DO), ammonia and CO _2_; and fish variables, particularly fish weight. These variables would allow the monitoring of the intra-pond environment itself and the fish biomass. Furthermore, non-observable variables such as fish appetite, fish anabolic and catabolic rates, fish oxygen consumption, among others, would be estimated.

On the next level, a prescriptive digital twin would anticipate future states and the behaviour of the fish cultivation unit. For example, times series of fish weight growth, release of biochemical species and consumption of oxygen would be predicted with the use of mathematical dynamic models combined with farm internal and external data.

In the sequence, a perspective digital twin would translate the information obtained from the prescriptive digital twin into real-time actionable suggestions, allowing operators to optimize feeding, oxygen supply and flow rates, and to enhance the control on released nitrogen in wastewaters.

The perspective digital twin assumes that decisions are taken by humans, who would then execute interventions either on site or remotely. On the other hand, the autonomous digital twin assumes that the decisions and controls are executed autonomously without human intervention, such as automatic valves that periodically adjust the supply of liquid oxygen according to automatically calculated demands for ensuring water quality and fish welfare.

### Target physical object

The physical objects to be emulated by the digital twin should correspond to objects required for the execution of the activities to be targeted by the twin. Since the core activity mirrored by the present twin will be fish growth cycles, an operational water basin will thus be the broad target physical object. A water basin is composed by the basin structure, associated systems (e.g., oxygen and feed deploy systems, sluice gates etc.), sensors, and the contents of the basin (water, fish biomass, feed, DO and other chemical species). Fish farms typically contain several basins, where fish growing cycles at different stages take place. In this work, association of basins either in series or in parallel in terms of fluxes of water or fish biomass will not be considered.

### The digital object

Compared to most industrial applications, including agriculture and livestock farming, aquaculture is characterized by a higher dependency on the ambient conditions. Also, dealing with subsurface environments is generally more demanding compared to terrestrial counterparts. Most aquaculture operations, such as feeding, act on the fishes via the water (an example of exception is individual fish vaccination), and these operations are typically targeted at the entire population of the tank, rather than on individuals or small groups. Furthermore, fish metabolism is highly dependent on water temperature and in case of unfavourable ambient conditions, such as temperature extremes, anoxia, presence of contaminants and pathogens, the entire population is affected.

A rigorous management of the environment is thus required for aquaculture farming, as decisions and actions typically impact large populations at once (normally 10 ^5^ to 10 ^6^ individuals in each of the basins of the pilot site study presented in Section 3). On this account, quantifying relationships between environmental variables and animal variables is a fundamental feature of the mathematical model that translates these variables into present and future descriptions of the digital object. The most important environmental variable is water temperature, since it acts as a forcing factor to several processes, e.g., fish respiration rate, fish metabolic rates (anabolic and catabolic), oxygen saturation rate. Other typical environmental variables include concentration of oxygen, ammonia and other chemical species, and pH. Animal variables include fish wight or size, number of stocked individuals, number of deaths, and several non-observable variables (e.g., fish respiration, fish metabolic rates). Parallel to environmental and animal variables, the digital representations will then be organized into two components: water and fishes, respectively.

To this extent, we have concentrated on providing a comprehensive and converging methodological approach, applicable to typical finfish rearing cycles conducted in inland aquaculture systems. To advance towards a conceptual application, it is required to specify an inner layer of details which varies among target systems, e.g., target finfish species, data acquisition systems, automatic and manual operations, mechanization of instruments, among others. The following section will then present a use case for a digital twin which is based on a real, operational trout farm.

## Use case

### The pilot site

The pilot site is a land-based trout farm located in the Trentino-Alto Adige region, Northern Italy. Because of the high availability of freshwater suitable for trout welfare, with low temperatures and high oxygen concentration, Northern Italy concentrates 80% of the farms and 75% of the mass production of trout in Italy (Castiglione *et al.*, 2009). In Northern Italy, trout farming is a well-established, traditional activity and the farming setting is typically made of small familial activities where the introduction of technological innovation is obstructed by a low economical investment ability. The pilot site is representative of the technology level of trout farming in Italy and thus constitutes an adequate use case to design innovation strategies that can support the farmer in their day-to-day decisions.

**Figure 1.**
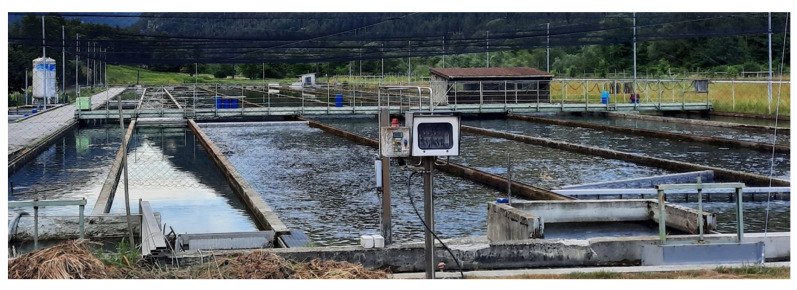
Raceway tanks, the liquid oxygen tank (white tank in the left) and the gantry (containing movable catwalk which crosses the raceways).

The fish growth from fingerlings up to the commercial weight takes place in six 200m-long and 8m-wide flow-through raceway basins ( Figure 1), which are covered by protection nets, in order to avoid bird predation. The influent quality varies in time, as the water is sourced from neighbouring Sarca river. The water receives a point source of oxygen upon entering the raceways, to fulfil the oxygen demand by fish respiration and to guarantee the water quality both in the raceways and the outflows back to the Sarca river. Liquid oxygen is stored in a steel tank and is supplied to each raceway independently via a network of pipes. The conversion of oxygen from liquid to gasified form takes place as the oxygen contacts the ambient temperature, at an efficiency rate of approximately 90%. The oxygen flow to each raceway is currently regulated through manual valves. The feed is deployed manually from a gantry, which during feeding is set in motion along the longitudinal direction of the raceways. Figure 1 shows the raceway tanks, the liquid oxygen tank (white tank in the left) and the gantry which contains the movable catwalk which crosses the raceways. The shed on the right is also part of the gantry and moves longitudinally along with the catwalk, as well as perpendicularly to the raceways. The company that manages the facility is associated the ASTRO Consortium ( https://www.troteastro.it/). ASTRO has developed a standardized protocol which covers key aspects of farm management, such as feeding, stocking density, water quality standards, quality of the final product (e.g. condition factor and flesh chemical-physical proper-ties). The compliance with the protocol requirements is certified by a Protected Geographical Indication (IGP) label.

The farm is equipped with sensors for real-time monitoring of water temperature and dissolved oxygen. In the pilot raceway, this data was collected from two identical multi-parametric automatic EXO2 sensors deployed near the upstream and downstream ends of the raceway. Data are processed and visualized using a dedicated software: at present, operators decide when to activate the oxygen supply on the basis of this information. There is an alarm system which is activated in the case of low DO concentration.

### The control structure of the digital twin

**Figure 2.**
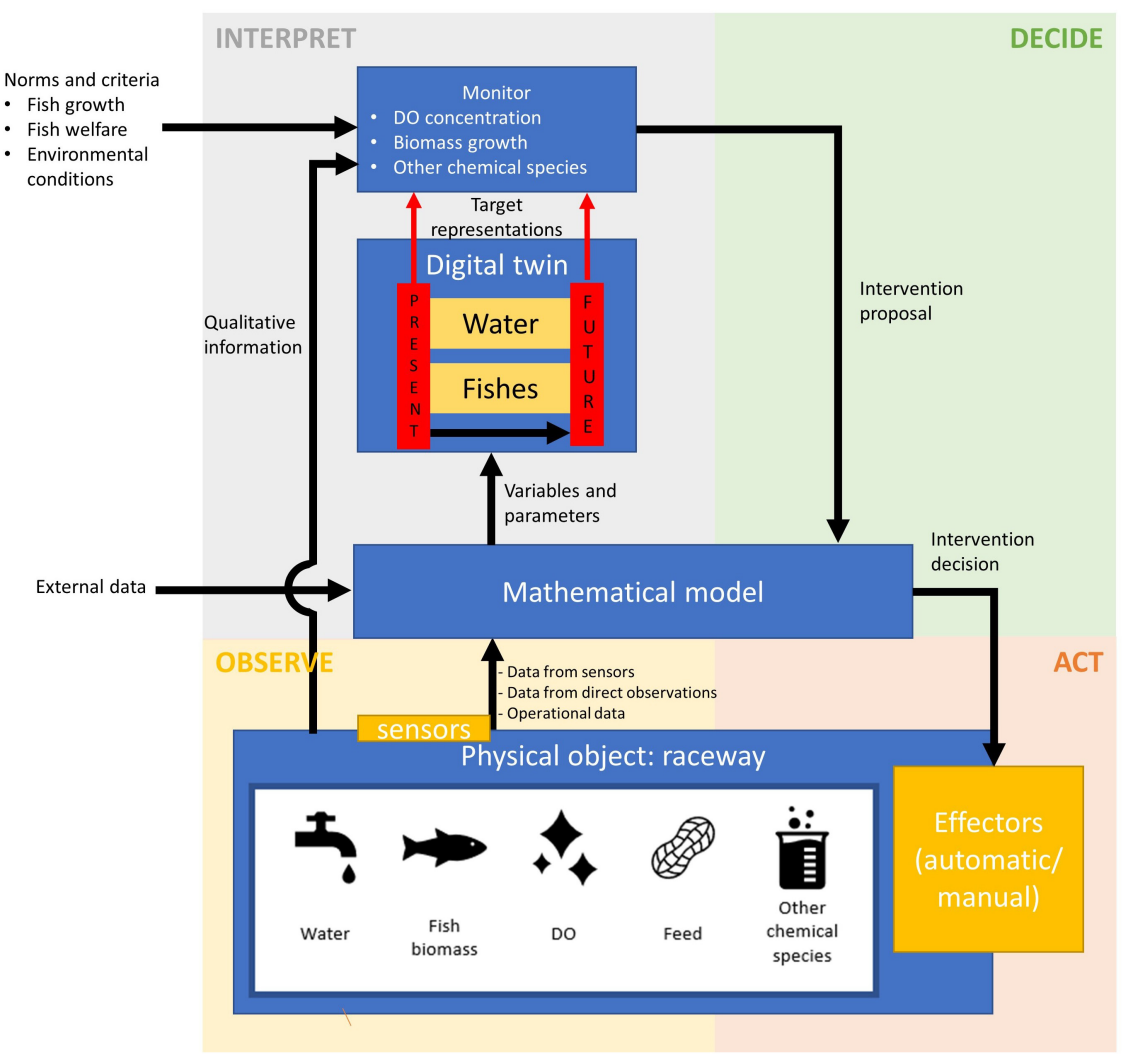
Information flow diagram for the digital twin conceptual model. DO, dissolved oxygen.

Figure 2 depicts the information flow diagram of the cyclic control structure of the digital twin, with control functions and information flows distributed along the four phases of farm management, i.e., observing, interpreting, deciding and acting. Each of these four phases in the context of the digital twin is described in the following subsections.

### The observing phase

The essential objective of the observing phase is to extract quantitative and qualitative information on the bio-responses of the fish, which is conducted via direct observations and data acquisition tools. The observing phase, along with the acting phase, is directly linked to the physical object. In a fully autonomous twin, the links between the physical objects and their information and data are decoupled from manual operations. In the present concept this is only partially realized, as it envisages manual inputs of data.

**Figure 3.**
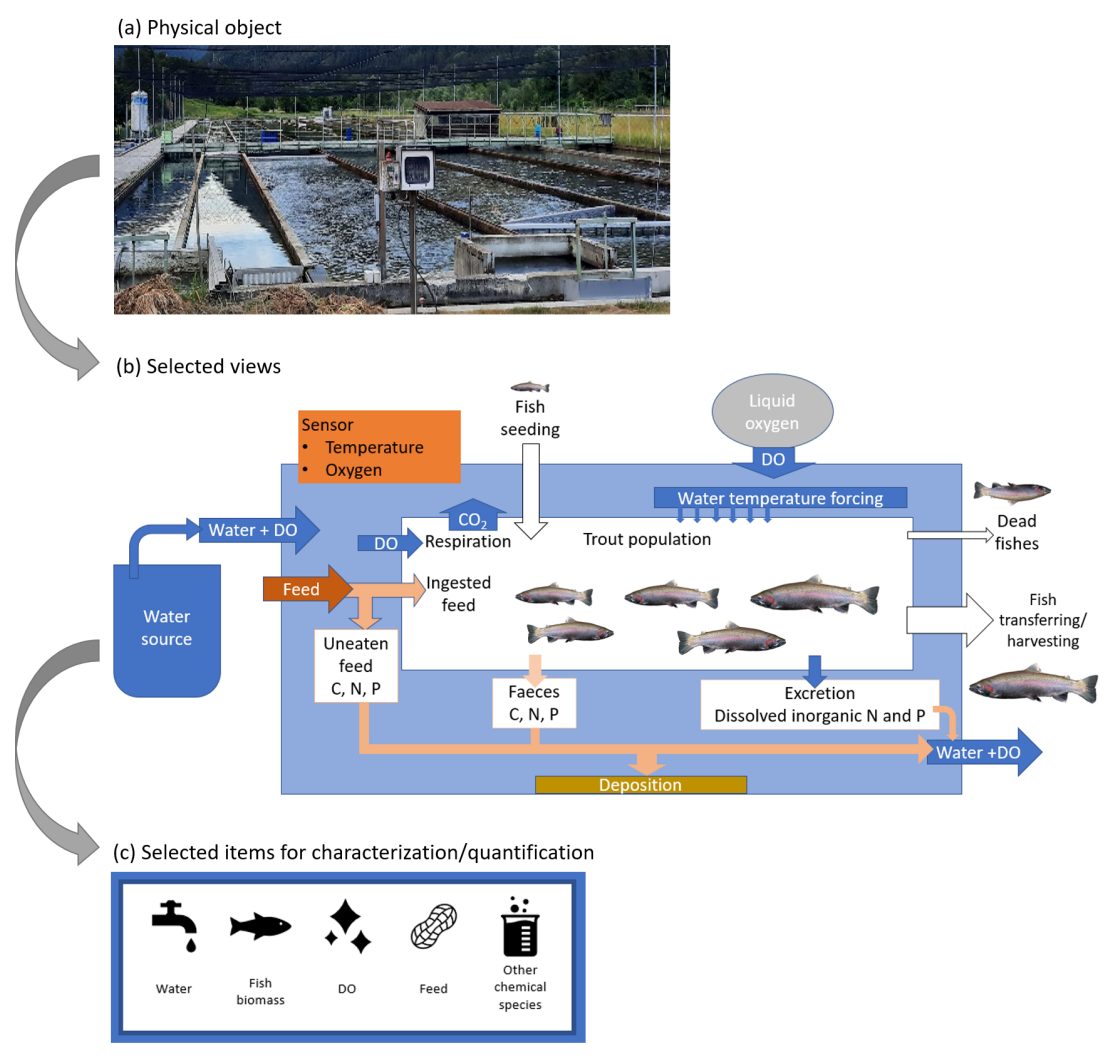
Delimitation scheme of the physical object. DO, dissolved oxygen.

Figure 3 shows the delimitation of the physical object, starting from a broad view of the system, i.e., merely the raceway (a). As a step further, the processes and physical entities which concern the fish bio-responses are selected, resulting in the view shown in Figure 3 (b). Finally, the view of the physical object that will be captured by the digital twin concerns the qualitative and quantitative description of the water, fishes, DO, feed and other chemical species. The view of the physical object is then reduced as the aggregation of these five components object, as depicted in Figure 3 (c). Appended to this reduced system, there are data retrieving systems, which constitute control units under the observing phase, and physical control systems, which concern the acting phase.

Qualitative characterization may include observation of fish behaviour, water colour and transparency and odour, and may be used in the interpreting phase, e.g., unusual fish behaviour may indicate diseases, particularly if high mortalities are observed along, and may require adaptations in the feed table. It is envisaged that as the digital twin concept evolves further, qualitative information that currently flows without being input into mathematical model ( Figure 2) will take the form of quantitative datasets to be incorporated into the model-based transformations. This may include for example the use of underwater cameras or sensors along with algorithms that identify diseases or parasites.

Direct fish observations include the periodical weighting of fishes, and the daily counting of the number of mortalities, which will result into quantitative datasets. A trial has been conducted ^18^ on the acquisition of fish weight using Biomass Daily (BD), an 80 × 80 cm submerged frame equipped with a sensor based on infrared technology which detects a signal whenever a fish specimen moves across the frame. To the authors’ knowledge, the application of BD to a trout raceway is novel. Sensors for temperature and DO are permanently deployed, usually positioned near the raceway inlet and outlet, and data is registered at regular intervals, normally every 15 min to 1 h. Concentration of ammonia and phosphate may be sporadically obtained via analysis of water samples. To complete the data sets, operational data is provided, specifically the supply rates of 1) water, 2) DO and 3) feed, and the characterization of feed composition.

Overlapping methods may be utilised for the acquisition of data corresponding to the same state variable. Examples in the present application include the estimation of fish weight by 1) direct weighting 2) using Biomass Daily and 3) using the mathematical bioenergetic model, or the determination of the DO concentration from 1) the measuring probes and from 2) the mathematical model for DO transport. By combining observations and model predictions, Data Assimilation (DA) schemes that update the accuracy of forecasts with observations using model-data filtering approaches may be integrated with the digital twin, as the one described by Royer *et al*. ^19^, which is targeted at the DO concentration dynamics and is capable of estimating the fish respiration.

### The interpreting phase

The farm model simulates the evolution of fish biomass and of a set of state variables connected with fish metabolism, and the numerical solution is obtained by coupling a module that simulates the evolution of fish biomass and a module that predicts those of a set of water quality variables affected by fish metabolism.

**Figure 4.**
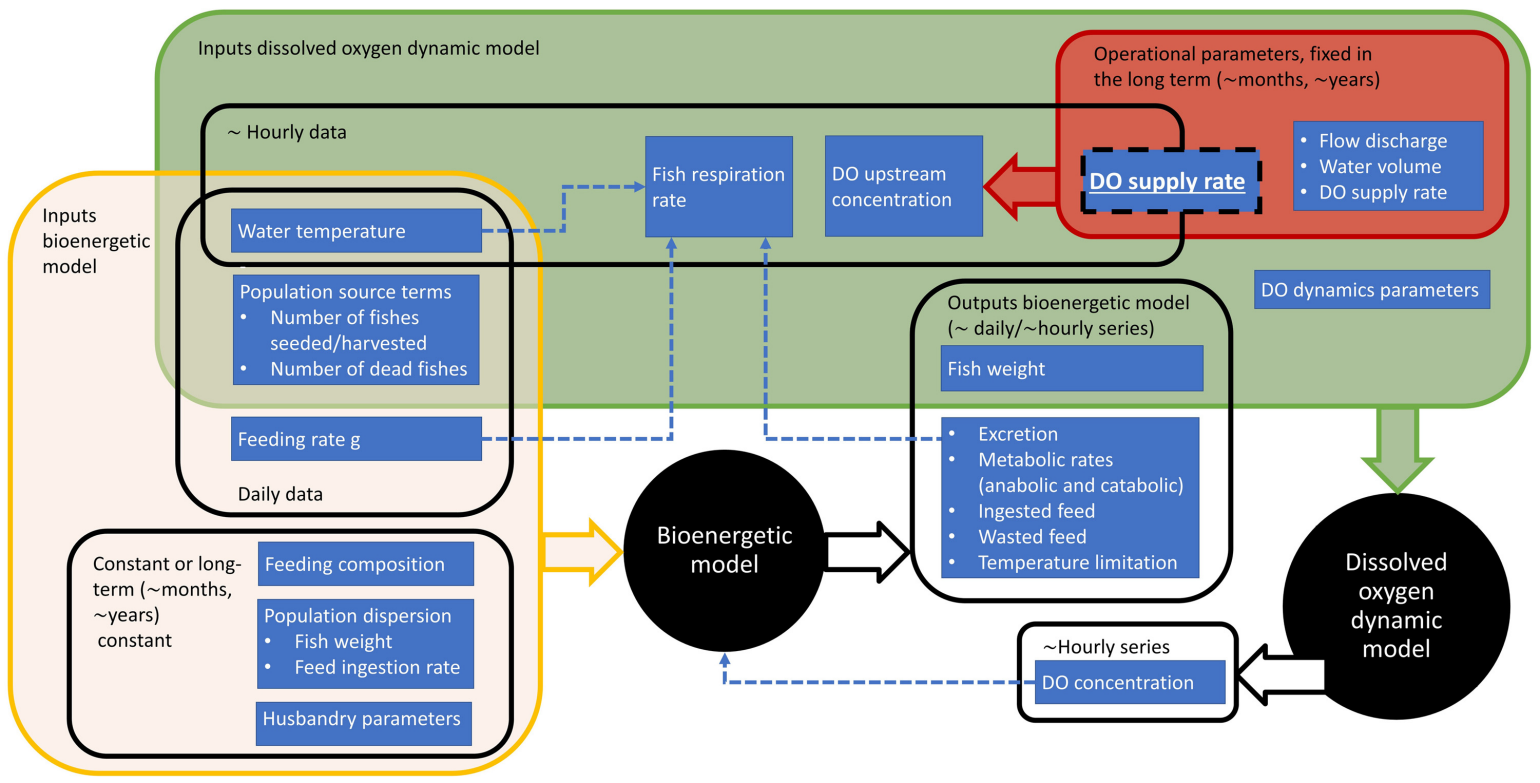
Scheme of inputs and outputs to the bioenergetic and DO (dissolved oxygen) dynamic models.

A core component of the digital twin control system is the mathematical model which unifies disparate data streams and provides real-time and forecasted pictures of the state of the physical object. In the present application, the mathematical model simulates the evolution of fish biomass and of a set of state variables connected with fish metabolism, and the numerical solution is obtained by coupling a bioenergetic module that simulates the evolution of fish biomass and a DO module that predicts the DO dynamics affected by fish metabolism ( Figure 4). The model is coded in R language version 4.1.0 (see *Software availability* ^20^).

The mathematical formulation of the bioenergetic model for rainbow trout is analogous to that described by Brigolin *et al*. ^21, 22^, Baldan *et al*. ^23^, developed for European seabass ( *Dicentrarchus labrax*) and gilthead seabream ( *Sparus aurata*), and considers that the weight change along time derives from the budget between the energy intake from fish feed and energy used for fish metabolism. The functionalities of the model for predicting the growth of rainbow trout have been recently validated ^24^. The bioenergetic model simulates the growth of rainbow trout according to water temperature, water quality and feeding regime, at both individual and population levels, as desired by the user. The effect of feeding into fish metabolism takes into account the feeding ration, hence the actual amount of feed provided by the fish farmer, and its biochemical composition in terms of content of crude proteins, crude lipids and carbohydrates. Water temperature affects both fish metabolism and ingestion. As shown in Figure 4, the bioenergetic model is run based on typically daily or hourly data for the forcings and accordingly returns sets of daily and hourly data.

The time series of fish biomass weight calculated from the bioenergetic model is input into the DO dynamic model ^18, 25^, with the aim to assess the rapidly changing DO concentration in the raceway basins. The interaction between the bioenergetic and DO dynamic mode The DO flux along the raceway streamwise direction is described by the advection equation with additional terms ex-pressing 1) the capture of oxygen via exchange with the atmosphere and 2) the oxygen consumption by fish respiration, the second and third terms on the right-hand side of Equation (1):


$$\frac{\partial C(x,t)}{\partial t}=-\frac{Q}{A}\frac{\partial C(x,t)}{\partial x}+k_{rear}\;(C_{sat}(x,t)-C(x,t))-\frac{R(t)}{A}\frac{\partial M(x,t)}{\partial x},\;(1)$$

where *x* is the streamwise coordinate (m), *t* is time (h), *C* is the DO concentration (mg L ^-1^), *Q* is the water flow rate (L h ^-1^), *A* is the raceway cross-sectional area (m ^2^), *C _sat_* is the DO saturation concentration (mg L ^-1^), *k _rear_* is the reaeration rate (h ^-1^), *R* is the oxygen consumption rate (mg h ^-1^ L ^-1^) and *M* is the fish biomass accumulated along the streamwise direction *x* (kg).

The indicative time intervals of one hour for the DO dynamic model scale with the typical water residence time in the raceways of the pilot site. Considering the inputs to this model, the water temperature, fish respiration rate and DO concentration at the water source assume sets of hourly values, as shown in Figure 4. The DO supply rate box shown in Figure 4 overlaps both short and long term time horizons. This depends on the frequency that the supply rate of the point source of liquid oxygen is adjusted. With a fully implemented autonomous digital twin, this supply rate should be adjusted in real time or minimally several times a day at a frequency that scales with water residence time in the raceway.

Apart from the total biomass and total oxygen consumption, which are directly explored by subsequent steps of the present twin concept, the bioenergetic model predicts the release of and metabolite excretions, i.e., ammonia and carbon dioxide. This latter capability of the model, currently underexplored by the present concept, can be integrated with environmental spatial data, economic-social criteria and policy issues ^22, 26^.

**Figure 5.**
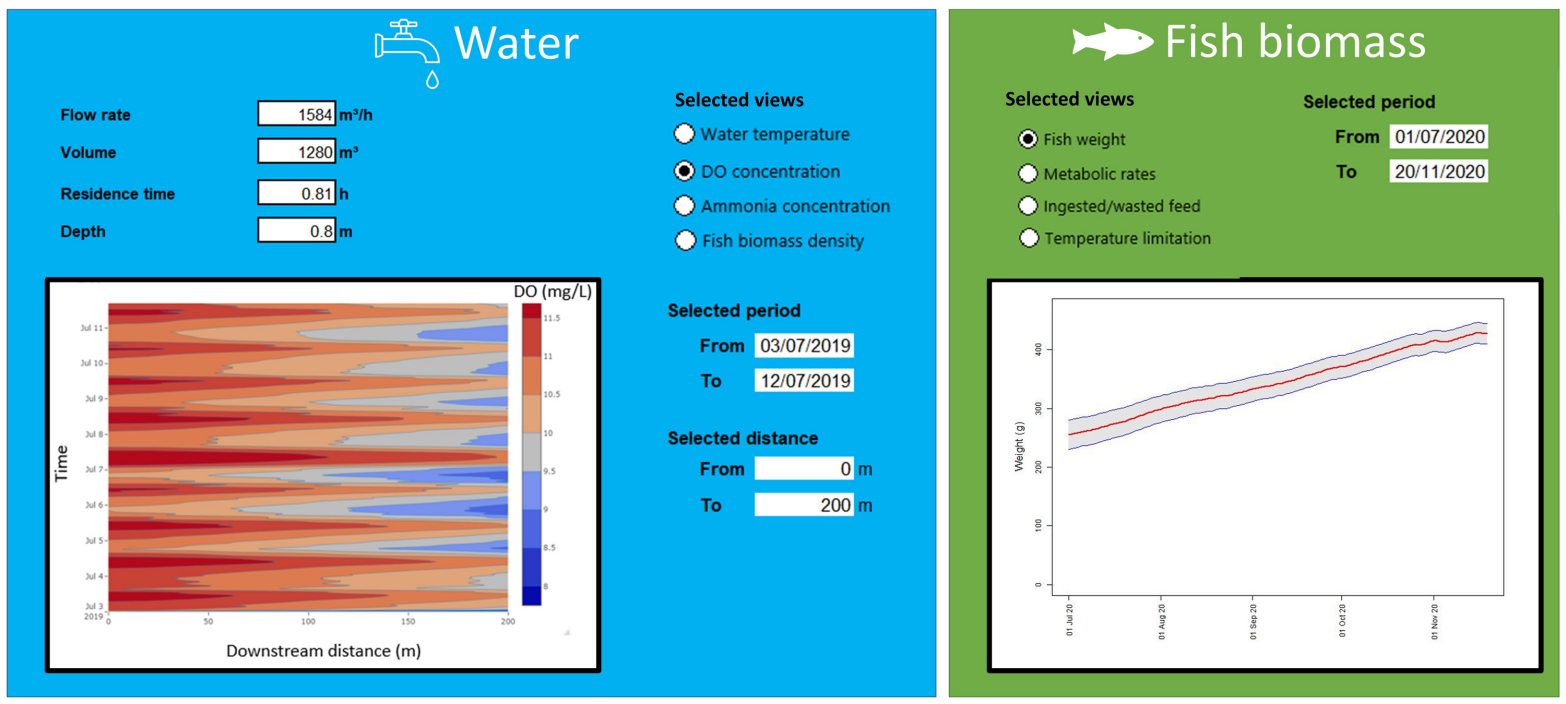
Selected views of the digital twin.

Upon securing the target datasets to describe the state of the system, the digital twin is constructed based on the organization of the datasets into the environmental (water) and animal (fish) components. The list of major variables for each component, along with illustrative views of the digital representations are shown in Figure 5. The water component shows a selected view of the DO concentration covering the full streamwise distance of the raceway (0 to 200m), from July 3 ^rd ^to 13 ^th^, 2019 (see *Underlying data* ^27^). The sinusoidal-like daily oscillation and the downstream decay of the DO concentration can be visualized. The fish view highlights the average fish growth and its standard-deviation bounds along a period of approximately 4.5 months.

Table 1 lists intervention proposals based on target monitored results. The decisions on executing these proposals take place in the deciding phase, detailed in the following subsection.

**Table 1.** Intervention proposals.

Intervention proposal	Based on	Typical frequency of execution
Harvest fish	Fish weight	year ^-1^
Transfer fish	Fish biomass density	month ^-1^, year ^-1^
Change type of feed	Fish weight	month ^-1^, year ^-1^
Define feed quantity for the next day	Fish weight	day ^-1^
Define liquid oxygen supply for the next hour	DO concentration	h ^-1^

### The deciding phase

**Figure 6.**
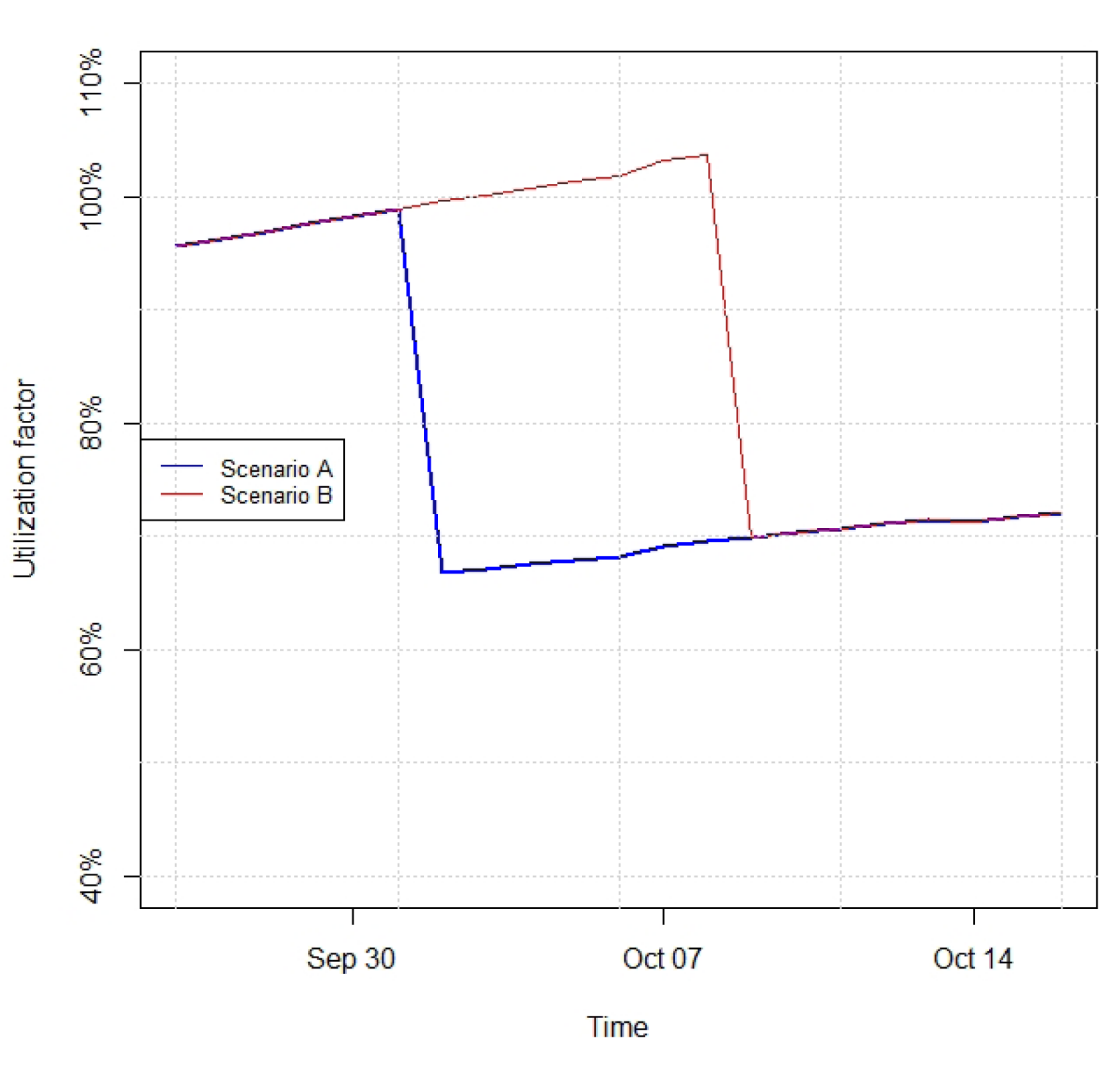
Utilization factor for Scenarios A (one-third of fish biomass transferred on October 1 ^*st*^) and B (one-third of fish biomass transferred on October 8 ^*th*^), envisaged based on different intervention proposals for fish transfer.

Based on intervention proposals from the previous interpreting phase, alternative future states of the digital twin are built using the mathematical model. Here we illustrate the case of transferring a fish cohort, which is to be executed based on the fish biomass density. The utilization factor presented in Figure 6 corresponds to the fish biomass density provided by the bioenergetic model divided by a baseline density for transferring fish of 20kg of fish per m ^3^ of water. The baseline density is set according to biomass transfer practices carried out at the pilot site and recommendations from Food and Agriculture Organization (FAO) ^28^. Figure 6 presents two scenarios (see *Underlying data* ^27^): A, in which the one third of the fish biomass is transferred one day before a utilization factor of 100% is reached, and B, in which the fish transfer of one-third of the total biomass is delayed one week compared to scenario A. As seen in the curves depicted in Figure 6, apart from the sharp decreases corresponding to the biomass transfer events, the biomass grows along time in both scenarios.

**Figure 7.**
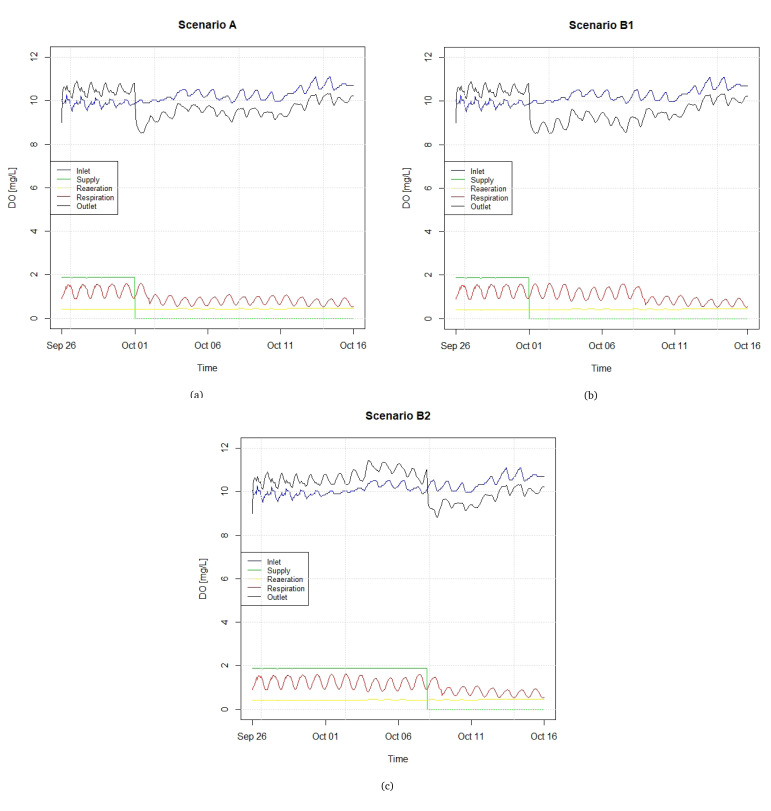
Components of the DO (dissolved oxygen) balance in different scenarios regarding fish transfer and DO supply.

To further illustrate the causal sequences of each scenario, Figure 7 shows the different components of the balance of DO under scenarios A and B, where the latter is subdivided into B1 and B2, based on alternative DO supply routines (see *Underlying data* ^27^). Figure 7 presents the components of the DO dynamic balance, specifically DO concentration in the raceway inlet and outlet, the point supply of DO, and the DO fractions associated with raeration and respiration, accumulated along the raceway streamwise direction (unlike the previous three components, which are point values, these are distributed along the raceway). The respiration represents a consumption, and is therefore a negative component of the DO budget. It can be observed that the DO at the inlet and the respiration component follow sinusoidal patterns, and, as a consequence, so does the DO at the outlet. In Scenarios A and B1, it is assumed that a constant supply of liquid DO of 2.4 m ^3^ h− ^1^, which after gasified adds to the DO budget a nearly constant value of approximately 2 mg L ^-1^, is interrupted on October 1st, whereas in Scenario B2 this supply is interrupted on October 8th. In line with the fish transfers expressed in Figure 6, punctual decreases in the respiration component can be visualized. In the three scenarios depicted in Figure ref Figure 7, the interruption in the supply of liquid oxygen causes the DO level at the outlet to be lower than in the inlet. The supply is cut in October because the metabolic rate of fishes decreases as winter approaches, and so does the demand of DO by the fishes.

The example presented in this subsection illustrates that multiple criteria are used for evaluating a proposed intervention, and finally decide which intervention will be executed.

### The acting phase

Currently, at the pilot farm, the supply controls for feed and oxygen are either fully manual or mechanized controls activated manually. This condition associated with decision supporting tools provided from the mathematical model would configure a digital twin of prescriptive level. To evolve towards the autonomous level, where operators are decoupled from the physical controls, the most plausible preliminary development in the pilot farm would be the installation of automatic valves to control the supply of liquid oxygen. To do so, and with reliable forecasts of fish respiration rate and DO concentration within the raceway, the control system must be converted from the current open-loop type to close-loop. In the open-loop case, the actual DO in the raceway has no input on the decision on the DO to be supplied, i.e., the supply of DO is, in principle, independent of the DO observed in the raceway. On the other hand, for a close-loop system, the valve is adjusted based upon the error between the actual DO and the desired DO. Therefore, the actual DO in the raceway has feedback on the control system.

**Figure 8.**
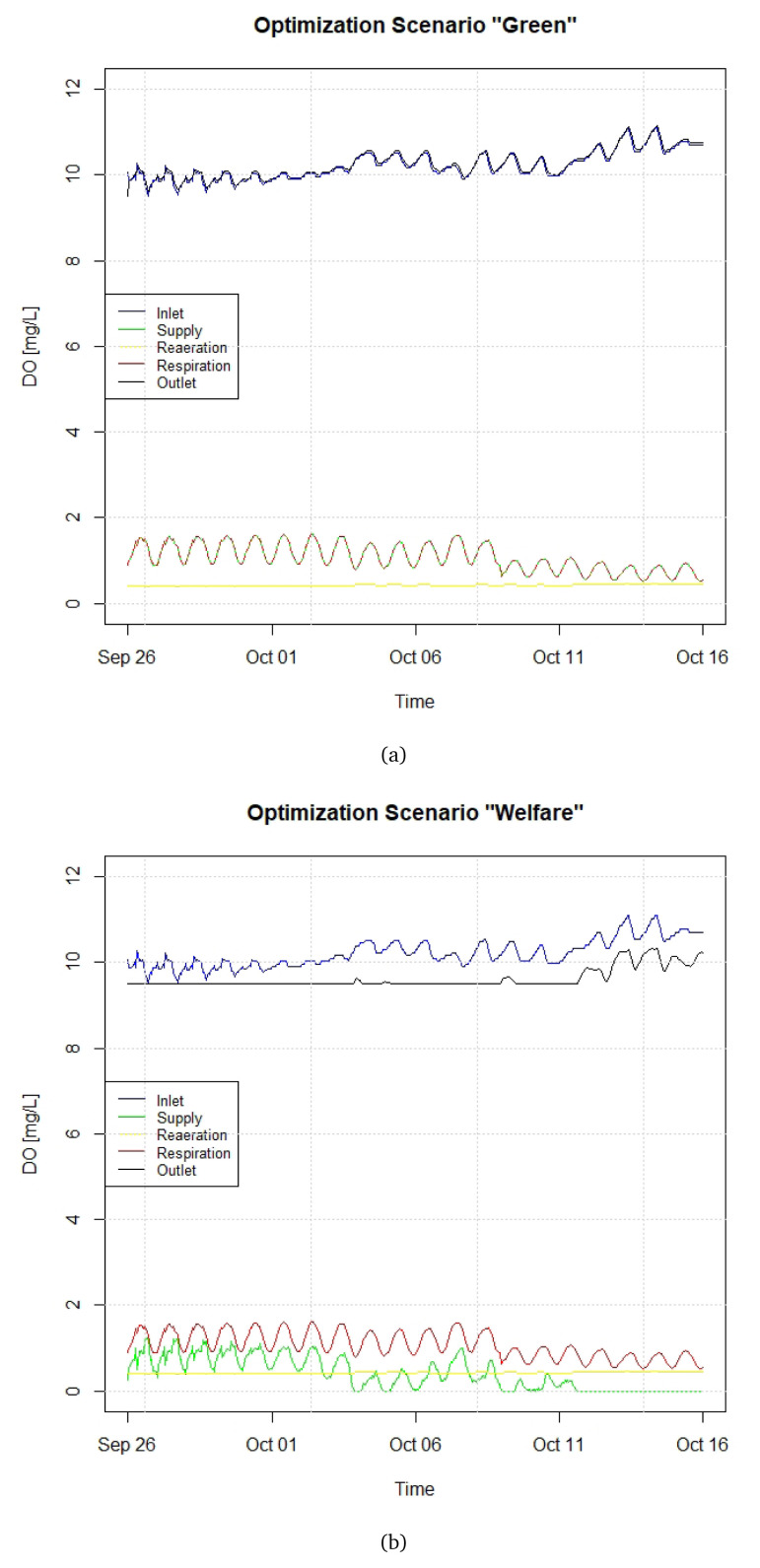
Close-loop control scenarios ( **a**) Green and ( **b**) Welfare. DO, dissolved oxygen.

Figure 8 depicts the components of the DO budget for two illustrative close-loop optimisation scenarios, referred as “Green” and “Welfare” (see *Underlying data* ^27^). Scenario Green ( Figure 8(a)) envisages an oxygen supply that returns to the stream water a DO concentration which is equal to the one at the raceway inlet. This means supplying a quantity of oxygen nearly coincident with fish demand. As such, this scenario ensures that the water returned to the stream (after the subtraction from fish respiration) will always present the same level of oxygen as in the inlet, and will then not affect the DO concentration of the stream. Scenario Welfare ( Figure 8(b)) guarantees that the DO concentration within the raceway does not to go below a welfare threshold, set as 9.5 mg L ^-1^. As such, this scenario ensures that the DO concentration will always remain above a welfare threshold for trouts, without taking into account the quality of the water returned to the stream. It should be more efficient from the oxygen supply point of view, and towards the end of the period shown in Figure 8(b), there is no need of extra DO supply.

In terms of fish biomass, both “Green” and “Welfare” are based on Scenario B of Section 3.5. By comparing the green lines of Figure 7 and Figure 8, it can be verified that the introduction of a close-loop control with an automated valve for DO supply would avoid an overconsumption of liquid DO such that the DO at the outlet is higher than at the inlet.

## Conclusions

In this paper, digital twin approaches employed in industry and agriculture were identified and combined with Precision Fish Farming techniques to build a conceptual digital twin for DO control, feed control and fish population management in a land-based recirculating aqua-culture system for rainbow trout. The methods aggregate solutions that need core science research, and provide a foundation for further research on precision aquaculture.

Among the several activities which compose the lifecycle of a fish farm or a fish rearing cycle, here the focus was on the production phase, specifically the growth of fishes from fingerling stage up to the commercial size. In parallel, the broad physical object to be mirrored by the digital twin was the recirculating aquaculture system, its equipment and contents. Owing to the high importance of the ambient environment in aquaculture systems, and because the state of these systems is described by means of environmental and animal variables, the digital object was organized into two components representing the water and the fishes.

It is expected that the pilot site represents a typical trout farm in Italy. The provided solutions are compatible with the practices and development level of these farms, and make use of mostly low cost and low maintenance sensors. Since the twin is envisaged for typical activities in a typical plant, it is expected that the methodology can be applied to other trout farms. Moreover, with the use a bioenergetic model targeted at other finfish species, the methodology can be transferred to other types of fish cultures.

The digital twin was built by aggregating several state-of-the-art developments on Precision Fish Farming, namely sensors and data acquisition systems, data assimilation, mathematical modelling and, as a further development, Internet of Things. Future developments may consider 1) the development of non-internet components, such as mechanical systems that will be integrated into the digital twin and enable remote operations, 2) the expansion to include boundary activities of the fish rearing cycle either in terms of the fish production cycle (e.g., fish seeding, fish storage after harvesting) or in terms of the plant itself (e.g., raceway design, acquisition of new equipment), and 3) a further expansion towards external logistics, including the incorporation of socioeconomic drivers such as human behaviours which influence the system and market networks.

## Ethics and consent

Ethical approval was obtained for the overarching Horizon 2020 GAIN Project, however the part of the study described in this paper did not deal with manipulation of animals or testing of fish feed, but only non-invasive monitoring systems. Thus, ethical approval for this study was not required.

## Data Availability

Zenodo: Underlying data - Digital Twin for Rainbow Trout ( *Oncorhynchus mykiss*) land-based aquaculture. https://doi.org/10.5281/zenodo.5691399 ^27^. This project contains the following underlying data: - Figure_5_DO_Concentration_x_t.csv. - Figure_6_Utilization_factor_Scenario_A_and_B.csv. - Figure_7a_Scenario_A.csv. - Figure_7b_Scenario_B1.csv. - Figure_7c_Scenario_B2.csv. - Figure_8a_Scenario_Green.csv. - Figure_8b_Scenario_Welfare.csv. Data are available under the terms of the Creative Commons Attribution 4.0 International license (CC-BY 4.0).

## Software availability

Source code available from: https://github.com/adrianocl/DTRT

Archived source code at time of publication: https://doi.org/10.5281/zenodo.5638971 ^20^

License: Mozilla Public License 2.0.
