# Supplementary material for: Digital twins for land-based aquaculture: A case study for rainbow trout ( Oncorhynchus mykiss)
Source: Open Res Eur. 2023 Nov 28;2:16. Originally published 2022 Feb 4. [Version 2] doi: 10.12688/openreseurope.14145.2 (PMC10472065; doi:10.12688/openreseurope.14145.2)
Supplement: Peer review history

# Reviewer report: Reviewer response for version 1

**DOI:** 10.21956/openreseurope.15250.r28802

**Published:** 2023-08-31

**Authors:** Murat Yigit

**Affiliations:** 1 Faculty of Marine Science and Technology, Department of Marine Technology Engineering, Canakkale Onsekiz Mart University, Canakkale, Turkey

I have evaluated the originality, significance and technical quality of the study, and the length and clarity of the review paper, which title is given below.

 Title: **“** **﻿** **Digital twins for land-based aquaculture: A case study for rainbow trout (Oncorhynchus mykiss)** **”**

 This is a very interesting and well documented paper, providing novel information on conceptual digital system that mirrors fish production activities in inland fish cultivation facilities, demonstrating quantitative processing of environmental data and biological responses for optimization of the feeding, oxygen supply, water quality and fish biomass increase. The study provides field testing in a case study based on a real operational activity in a Rainbow trout ( *Oncorhynchus mykiss*) farm based in Italy.

 Based on the information provided, it is well understandable that the system introduced, is a pioneer application towards supporting fish farmers for optimization of feeding practices, oxygen supply in ponds and biomass control and monitoring and population management with regards to growth performance of fish, welfare, and environmental loads from fish farm effluents. The digital twin system applied here, uses data obtained from sensors by estimated mathematical data modeling, which are as novel developments in the precision of dish cultivation strategies with real applications in land-based aquaculture facilities in a case study on rainbow trout as the selected finfish species. Based on the implemented system, it has been underlined that this system enables real-time and remote management, as well as the reproduction of real and forecasted scenarios for a land-based trout farm.

 In conclusion, it has been underlined that the digital twin system was built through gathering several state-of-the art developments on Precision Fish Farming, namely sensors and data acquisition systems, data assimilation, mathematical modelling, using internet support. Future developments have been encouraged to consider the development of non-internet components, such as mechanized feed silos, the expansion to include boundary activities of the fish rearing cycle either in terms of the fish production cycle or in terms of the facility itself, and also a further expansion towards external logistics, including the incorporation of socioeconomic drivers such as human behaviors which are closely linked to the system and market networks.

 In brief, the paper is well organized and comprehensively described, and the rationale for developing the new method is clearly explained. The method description is technically sound, and details are sufficiently provided to allow replication of the method development and its use by others. Results are presented so that reproducibility could be easily performed. The conclusions about the method and its performance are adequately supported by the findings presented in the article.

 I may recommend to accept the article in its present form without any further improvements.

Is the rationale for developing the new method (or application) clearly explained?

Yes

Is the description of the method technically sound?

Yes

Are the conclusions about the method and its performance adequately supported by the findings presented in the article?

Yes

If any results are presented, are all the source data underlying the results available to ensure full reproducibility?

Yes

Are sufficient details provided to allow replication of the method development and its use by others?

Yes

Reviewer Expertise:

Aquaculture and Environmental Interactions

I confirm that I have read this submission and believe that I have an appropriate level of expertise to confirm that it is of an acceptable scientific standard.

# Response

**Published:** 2023-10-25

**Authors:** Adriano Lima

**Affiliations:** Ca' Foscari University of Venice, Italy

Dear Murat Yigit On behalf of the authors, I would like to thank you for your effort in handling this manuscript. I highly appreciate the kind recognition of the merit of our study, as well as the insightful comments. Kind regards Adriano Lima

# Reviewer report: Reviewer response for version 1

**DOI:** 10.21956/openreseurope.15250.r28956

**Published:** 2022-09-01

**Authors:** David Jones

**Affiliations:** 1 University of Aberystwyth, Aberystwyth, UK

This paper presents an implementation of the digital twin in the use-case of precision fish farming. The paper is well-written, it is an interesting read, and from a digital twin perspective, I have no issues recommending its approval.

 Reviewing from a digital twin perspective, the paper is sound in its interpretation and implementation of the digital twin. The thought and effort that has gone into the selection of the key parameters of the use-case and their inclusion into a mathematical model is good to see.

 I have some very minor comments:

1.  “external data” is referred to, can a description of this be added please?
2. In Figure 3, from left to right, the fish get bigger and are swimming against the direction of the arrows in the diagram. Is this deliberate? My sense is all fish should be swimming from left to right too.
3. The conclusion mentions “non-internet components” and gives an example of a mechanized feed silos. I am not clear by what is meant here, are you thinking about internet-enabling these components such that they can be included in your digital twin? It might be worth re-writing this bit to be clearer.
4. Given the use-case does not align with my background, there were a few terms that were new to me. If you did want to broaden the appeal of the paper, some form of map/diagram/descriptions of the site and the activities would help.
5. Grieves talks about the twinning of both the entity and environment, this is an interesting point in terms of your work. Whether the fish is the entity, and the farm/surrounding is the environment, or whether the farm (including the fish) is the entity, and the wider surroundings being the environment. The paper doesn’t need to be changed to account for this, but it might be worth consideration in your future work.

Finally, I’m only able to review this paper from a digital twin perspective, I would recommend it be reviewed by someone with expertise in fish farming.

Is the rationale for developing the new method (or application) clearly explained?

Yes

Is the description of the method technically sound?

Yes

Are the conclusions about the method and its performance adequately supported by the findings presented in the article?

Yes

If any results are presented, are all the source data underlying the results available to ensure full reproducibility?

Yes

Are sufficient details provided to allow replication of the method development and its use by others?

Yes

Reviewer Expertise:

My area of expertise is in the digital twin. The paper needs reviewing by someone with expertise in fish farming.

I confirm that I have read this submission and believe that I have an appropriate level of expertise to confirm that it is of an acceptable scientific standard.

# Response

**Published:** 2023-10-25

**Authors:** Adriano Lima

**Affiliations:** Ca' Foscari University of Venice, Italy

Dear David Jones On behalf of the authors, I would like to thank you for your effort in handling this manuscript. I highly appreciate the kind recognition of the merit of our study, as well as the insightful comments. The manuscript was revised with attention to your recommendations. Please find below point-by-point responses to the comments.

- “external data” is referred to, can a description of this be added please?

Answer: Answer: The word farm was added.

- In Figure 3, from left to right, the fish get bigger and are swimming against the direction of the arrows in the diagram. Is this deliberate? My sense is all fish should be swimming from left to right too.

Answer: The figure was modified to include fishes swimming not necessarily in a preferential direction.

- The conclusion mentions “non-internet components” and gives an example of a mechanized feed silos. I am not clear by what is meant here, are you thinking about internet-enabling these components such that they can be included in your digital twin? It might be worth re-writing this bit to be clearer.

Answer: The text was modified accordingly. Non-internet components need their own developments, and they are indeed aimed to be integrated into an internet-enabled system.

- Given the use-case does not align with my background, there were a few terms that were new to me. If you did want to broaden the appeal of the paper, some form of map/diagram/descriptions of the site and the activities would help.

Answer: Further information on the site and its management protocols were added.

- Grieves talks about the twinning of both the entity and environment, this is an interesting point in terms of your work. Whether the fish is the entity, and the farm/surrounding is the environment, or whether the farm (including the fish) is the entity, and the wider surroundings being the environment. The paper doesn’t need to be changed to account for this, but it might be worth consideration in your future work.

Answer: I appreciate the comment. Indeed, defining what is to be set as the entity can bring up a deeper discussion.
